# Biological 2,4,6-trinitrotoluene removal by extended aeration activated sludge: optimization using artificial neural network

**DOI:** 10.1038/s41598-023-34657-z

**Published:** 2023-06-03

**Authors:** Hossein Karimi, Farzaneh Mohammadi, Saeed Rajabi, Amir Hossein Mahvi, Ghader Ghanizadeh

**Affiliations:** 1 Health Research Center, Baqiyatallah University of Medical Sciences, Tehran, Iran; 2 Department of Environmental Health Engineering, School of Health, Isfahan University of Medical Sciences, Isfahan, Iran; 3 Student Research Committee, School of Health, Shiraz University of Medical Sciences, Shiraz, Iran; 4 Department of Environmental Health Engineering, School of Health, Shiraz University of Medical Sciences, Shiraz, Iran; 5 Center for Solid Waste Research, Institute for Environmental Research, Tehran University of Medical Sciences, Tehran, Iran

**Keywords:** Environmental sciences, Environmental social sciences

## Abstract

Serious health issues can result from exposure to the nitrogenous pollutant like 2,4,6-trinitrotoluene (TNT), which is emitted into the environment by the munitions and military industries, as well as from TNT-contaminated wastewater. The TNT removal by extended aeration activated sludge (EAAS) was optimized in the current study using artificial neural network modeling. In order to achieve the best removal efficiency, 500 mg/L of chemical oxygen demand (COD), 4 and 6 h of hydraulic retention time (HRT), and 1–30 mg/L of TNT were used in this study. The kinetics of TNT removal by the EAAS system were described by the calculation of the kinetic coefficients K, Ks, Kd, max, MLSS, MLVSS, F/M, and SVI. Adaptive neuro fuzzy inference system (ANFIS) and genetic algorithms (GA) were used to optimize the data obtained through TNT elimination. ANFIS approach was used to analyze and interpret the given data, and its accuracy was around 97.93%. The most effective removal efficiency was determined using the GA method. Under ideal circumstances (10 mg/L TNT concentration and 6 h), the TNT removal effectiveness of the EAAS system was 84.25%. Our findings demonstrated that the artificial neural network system (ANFIS)-based EAAS optimization could enhance the effectiveness of TNT removal. Additionally, it can be claimed that the enhanced EAAS system has the ability to extract wastewaters with larger concentrations of TNT as compared to earlier experiments.

## Introduction

Water quality and resources scarcity are two problems facing the world today due to the geographical, anthropological and socioeconomic influences^1^. Accordingly, sustainable recovery and reuse of the wastewater resources have long been considered as the promising approaches to overcome these problems. However, the presence of a broad range of hazardous compounds in wastewater, comprising pesticides, polycyclic aromatic hydrocarbons, polyphenolic compounds, oil, surfactants, and nitroaromatics, have made it more difficult to develop strategies with desirable efficiency to remove these contaminants^2,3^. With regard to many organic and inorgan compounds in domestic and industrial wastewaters, different biological and chemical wastewater treatment processes have been applied to overcome the pollution removal from wastewaters^4–8^.

Among the afore-mentioned contaminants nitroaromatic compounds (NAC) are considered as one of the major sources of the contamination in wastewater resources that can induce serious problems if not removed properly^9^. These compounds are a particular class of aromatic molecules with an at least a nitro group (–NO_2_) at their benzene ring. It has been demonstrated that NACs have widely been distributed in the environment^10^, and 1,3,5-trinitrobenzene (TNB), 2,4,6-trinitrotoluene (TNT), trinitrophenol, tetryl nitramine, hexanitrobenzene are among the well-known NACs^1^.

TNT (2,4,6-trinitrotoluene) is a multifunctional aromatic substance used in wide variety of fields including medicines, insecticides, fungicides, herbicides, polyurethane foams, and dyes. Besides, it is among the most traditional explosives still utilized in mining industry^11^. The TNT production process encompasses several steps generating waste materials that are eventually released into the surrounding environment and could potentially contaminate water resources and soil. Mono-nitro toluene (MNT), di-nitro toluene (DNT), sulfates, dinitro toluene sulfonate (DNTS), and various other nitrobenzenes (NB) are also the major derivatives of the TNT with potential health risks. It is well-known that varying quantities of these nitrogenous substances are present in TNT-contaminated wastewater and responsible for the discoloration (e.g., orange water, red water, yellow water, etc.)^9^. TNT-polluted wastewater has also been reported with high chemical oxygen demand (COD) levels, varying from 600 to 6000 mg/L^12^. In accordance, the presence of TNT and other related aromatic chemicals in wastewater were shown to massively affect the regional ecology. Based on the Environmental Protection Agency's (EPA) guidelines, the maximum concentration of TNT in water and soil should not exceed 2 µg/L and 17.2 mg/L, respectively^13,14^. Others have also shown that the presence of more nitrobenzene derivatives—MNT, DNT, sulfonates, and others- could increase the contamination levels^11–15^. Considering these notes, TNT-contaminated wastewater should be treated properly before its discharge into waterways.

Physical, chemical, and biological strategies have all been used in various treatment processes up to this point to eliminate or adsorb this wastewater pollutant^16^. Despite some of their benefits, they can lack cost-effectiveness or dependability for usage on a broad scale^17^. But in this situation, biological treatment methods that employ microbial consortia have received a lot of attention since they have the ability to mineralize TNT and generate harmless byproducts (microbial biomass, H_2_, and CO_2_). Due to their accessibility, high effectiveness, and environmental friendliness, biological methods have lately gained a lot of interest in the treatment of such wastewater. Additionally, effective biodegradation of these contaminants was possible under both aerobic and anaerobic conditions^18,19^.

Activated sludge process efficiency has been increased by a number of changes that have been made in accordance with the literature. Extended aeration-activated sludge (EAAS) is the most widely used modification of the activated sludge (AS) system among these, but using this method for wastewater treatment is subject to significant limitations like high retention time (HRT), low active biomass, and low organic loading rate. Contrarily, this system's significant aeration capacity and the container contents' complete mixing increase the process's dependability^20^. Moreover, low BOD outflow, low residual activated sludge, and low ammonia discharge are among the advantages of the EAAS. Therefore, since the advantages of the EAAS system are often exceeded its disadvantages, thus it seems to be essential to adjust the condition under which the best removal efficiency could be^21^.

Over the last decade, several mathematical modeling have been introduced to optimize the treatment processes. In this regard, artificial intelligence (AI) has widely been utilized for data simulating purposes as well as accurate optimization of the biological processes. Among the most commonly used AI approaches are adaptive neuro-fuzzy inference system (ANFIS), fuzzy logic, support vector machine, genetic algorithm (GA), response surface methodology (RSM), and Artificial Neural Network (ANN)^22^. The adaptive neuro-fuzzy inference system (ANFIS) is a modern and effective approach to modeling input–output relationships in complex systems^23^. In this method, it is possible to learn from training data as an ANN and then solve it on a fuzzy inference system (FIS). Finally, the hidden layers are identified exactly by a FIS in the ANFIS network. This method eliminates the important challenge of determining and predicting the hidden layers in the ANN model. This can be a strong reason for using the ANFIS method, while this approach does not have a complex mathematical model and is a fast and flexible method for developing predictive models of biochemical treatment processes^24^. A genetic algorithm (GA) is also a research method to obtain accurate or approximate solutions to optimization problems^24^. GAs are optimization techniques that are controlled based on the principles of evolution and natural genetics^24^. One of the advantages of this method is its ability to create clear models for complex and difficult systems. As a result, this method can be employed for optimizing systems in which discontinuous, random, and nonlinear functions are not suitable for standard optimization patterns^24^.

ANN is a common machine learning approach that is a subset of AI. Since knowledge of the physical characteristics of the process is not necessary, neural networks belong to the category of "black box" models. It establishes an association between the input and output variables. As shown by the rise of research over time, the tendency appears to be moving further in the direction of ANN models. This is conceivable since ANN offers several benefits over traditional models. These benefits include modeling complex non-linear functions with high precision, supporting multiple inputs and multiple outputs (MIMO) modeling, working with chaotic and imperfect data, requiring less processing effort, and allowing the model to be updated or trained using fresh data. Along with its benefits, ANN modeling has some drawbacks as well. These drawbacks include the fact that the model parameter (number of nodes, hidden layers) has no physical meaning and that there is no accepted method for determining the network design. Additionally, tests and faults can lead to overfitting or underfitting and do not offer a singular solution. Additionally, a poorly trained network may congregate to a local minimum. As a result, ANFIS and GA techniques were used in this work to simulate, model, and optimize the data. In addition to modeling the system's ideal performance and determining its kinetic coefficients, the aim of this study was to determine if the extended aeration biological system can successfully remove TNT from wastewater. There has not been any research on TNT elimination using EAAS, according to the evaluation of the literature.

## Materials and methods

### EAAS pilot experiments

**Figure 1:**
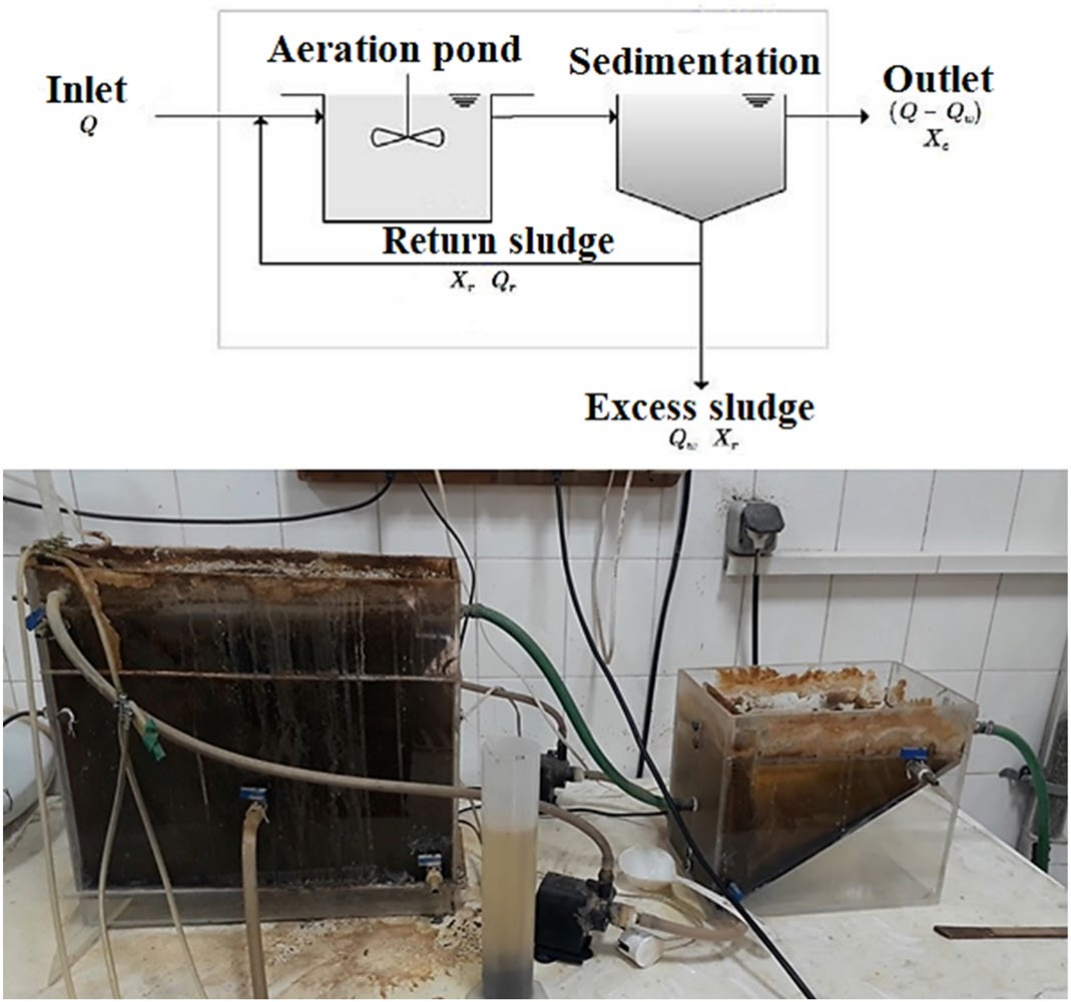
Schematic of EAAS pilot.

The experiment's materials, which included potassium dichromate, silver sulfate, mercury sulfate, acetonitrile, methanol, Triton x-114, and acetone, were bought from Merck in Germany. On a pilot scale, activated sludge with an extended aeration system comprising an aeration tank, a secondary sedimentation or clarifier tank, synthetic wastewater preparation, and collecting tanks was employed in this study. The aeration tank was 38.5 cm in height, 15 cm in width, and 44 cm in length, and the sedimentation tank was 21 cm in height, 20 cm in width, and 30 cm in length (Fig. 1). The capacity of the extended aeration tank was 34 L. The system was equipped with an effluent injection and sludge return pumps that operated continuously. The synthetic wastewater with COD (500 mg/L), HRT (4–6 h), and TNT (1–30 mg/L) was used in this study. In details, the synthetically prepared wastewater was increasingly added to the system until the equilibrium was attainted. Afterward, the concentration was increased to complete the process. Then, the remaining TNT in the effluent was analyzed using a high-performance liquid chromatography system (HPLC, Waters Co., and Millennium software) equipped with a UV–Vis detector model 486 and a used column (250 mm × 4.6 mm × 5 µm). Acetonitrile and water in a ratio of 80:20 served as the mobile phase. The injection volume was 20 µL, the flow rate through the column was 1 mL/min, and the absorption wavelength utilized was 210 nm. A sample of the extracted sample containing the explosive material was obtained every two weeks for COD testing, and analysis was carried out in accordance with the testing procedure described in the Standard Methods^11^. Finally, the process efficiency was calculated by the following equation:

1 $$ Removal\;\;Efficiency \, = \frac{{C_{0} { } - C_{t} { }}}{{C_{0} }} \times 100 $$

where *C*_*0*_ and *C*_*t*_ (mg/L) are the initial and final TNT concentrations^25^.

### Statistical analysis

SPSS-v.20 (Inc., Chicago, USA) software was used to perform statistical analysis. The Kolmogorov–Smirnov test was used to evaluate data distribution. The median and Mean ± SD were calculated.

### ANFIS model

The ANFIS model is based on the Sugeno structure with a fuzzy neural modeling created in MATLAB 2017 software. The ANFIS architecture is based on the artificial neural networks and fuzzy logic that obtains the optimal distribution of membership functions between input and output data. The five main layers of ANFIS model formation are (1) fuzzy layer, (2) product layer, (3) normalized layer, (4) non-fuzzy layer, and (5) total output layer.

In this study, several different membership functions such as triangular membership function (trimf), trapezoidal membership function (trapmf), generalized bell membership function (gbellmf), Gaussian curve membership function (gaussmf), Gaussian hybrid membership function (gauss2mf), P-shape membership function (pimf), the difference between two sigmoid membership functions (dsigmf), the product of two sigmoid membership functions (psigmf) were studied. To identify the most appropriate membership function, the data set obtained (110 samples) from the reactor was randomly divided into two categories: 85% training and 15% testing. Also, a hybrid learning algorithm was employed in this research to increase the convergence rate. This algorithm was used to integrate the two methods of least squares and gradients to update the input parameters. The least squares method was employed to optimize the input parameters. Then, the descent gradient method was applied to better adjust the default parameters. Finally, the ANFIS output was determined by the following parameters^26^. For this purpose, F/M, HRT, SRT, MLSS, MLVSS, SVI, and TNT_in_ were introduced as input parameters to the model. The removal of TNT was considered as the response parameter.

Of note, the accuracy and adequacy of the models presented by ANFIS were determined by calculating coefficient (*R*^2^) and root mean square error (*RMSE*) as follows (Eqs. 2 and 3)^27,28^:

2 $${R}^{2}=\frac{1}{N}\frac{\sum ({Y}_{prd,i}-{Y}_{prd,m})({Y}_{exp,i}-{Y}_{exp,m})}{{\sigma }_{prd}{\sigma }_{exp}}$$

3 $$RMSE=\sqrt{\frac{1}{N}\sum_{i=1}^{N}{({y}_{prd,i}-{y}_{exp,i})}^{2}}$$

### GA optimization

The GA was employed to obtain the optimal conditions for TNT removal as fitness function based on the ANFIS model developed in this study. All the GA analysis were performed in MATLAB software R2017a version.

$$ {\text{Fitness }}\;{\text{function}}\, = \,{\text{Max }}({\text{Removal}}\;{\text{ rate }}\;{\text{from}}\;{\text{ ANFIS\,simulation}}). $$

The other parameters considered in the genetic algorithm were: the number of generation 250, the Rank scaling function, the selection function of Stochastic uniform, the number of elite 2, the Crossover fraction equal to 0.8, and the mutation function of Constraint dependent and combination of Scattered function^28^.

## Results and discussion

### Effect of various parameters on EAAS performance

#### Effect of initial concentration of TNT

**Figure 2:**
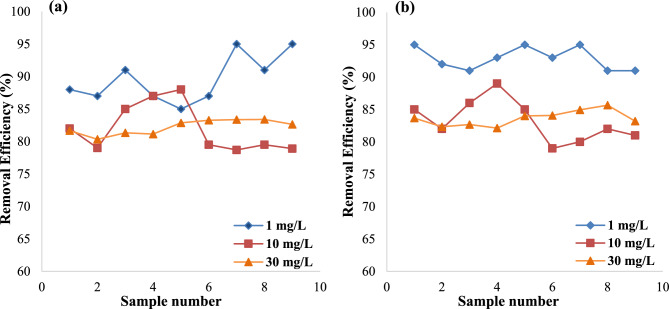
Comparison of TNT removal efficiency at different HRT 4 h (**a**) and 6 h (**b**) and TNT concentrations.

It is well known that exposure to a concentration of pollutants exceeded the limitation levels could affect the functioning and removing efficiency of the biological processes. In this study multiple concentrations of TNT (1, 10, and 30 mg/L) were used to assess the system removing efficiency during 4 and 6 h. As it is shown in Fig. 2a and b, the removal efficiency was declined by increasing TNT concentration from 1 to 30 mg/L. The average TNT removal values for 1, 10, and 30 mg/L were 89.56% ± 3.29, 80.43% ± 4.54, and 82.79% ± 1.43 at 4 h, and 92.83% ± 1.58, 82.08% ± 3.53, and 84.25% ± 1.93 at HRT 6 h, respectively. It can be assumed that this might be due to the toxicity of the pollutant in the process reactor or the generation of more hazardous byproducts, which prevents the effective removal of TNT by bacterial consortia. On the other hand, as TNT is nitrogenous by nature, it can also be inferred that higher concentrations of TNT influences the carbon source available for the bacteria, which in turn diminished the removal efficienc^16^. Permatasari et al.^30^ and Dionisi et al.^29^ have also shown that the ratio of C:N:P (100:5:1 to 100:10:1) is crucial in biological activities. Based on the results, the concentration of the 10 mg/L TNT was correspondent to the lowest removal efficiency; however, by increasing its concentration to 30 mg/L, the removal efficiency was increased. This might be interpreted by higher growth of the biomass in the reactor at higher pollutant concentrations that leads to the increased removal of TNT. Besides, at higher concentration of TNT, the time to adaptation of the biomass bacteria would be decreased, which in turn could be associated with higher removal of the investigated pollutant. Such arguments can also be true for lesser concentrations, as we observed the higher removal efficiency at 1 mg/L of TNT. Moreover, at lower concentrations the biomass bacteria are not exposed to higher levels of toxic byproducts^29,31,32^.

#### Effect of HRT

As shown in Fig. 2, the removal efficiency was increased by the increment of the hydraulic retention duration. It can be inferred that at higher retention time, bacteria could effectively break down the TNT compound, which then allowed more dead bacteria and active sludge to adsorb it into the surface. Moreover, more collisions and interactions may occur between TNT molecules and other chemicals in wastewater by increasing the hydraulic retention time that leads to higher removal of TNT molecules. Several other studies have also shown that more bacteria could adapt to the pollutant at longer retention time that would increase its elimination. Furthermore, a variety of chemicals or their newly formed byproducts in the wastewater may also increase the removal of TNT. Last but not the least; longer retention time gives the bacteria this opportunity to degrade the compound of interest more effectively^32,33^.

#### Removal efficiency of COD

**Figure 3:**
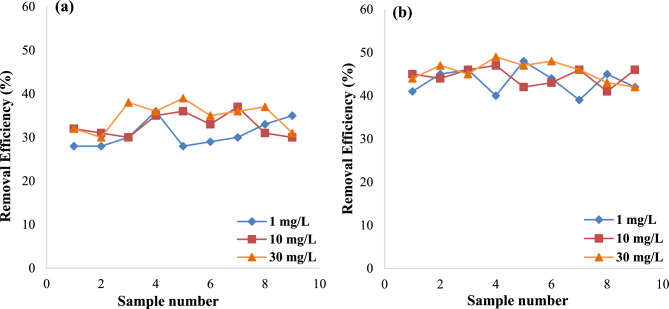
Comparison of COD removal efficiency at different HRT 4 h (**a**) and 6 h (**b**) and TNT concentrations.

As mentioned earlier, the concentration of pollutants can affect biological processes, which in turn affects the COD levels and associated process efficiency. As shown in Fig. 3a and b, by increasing TNT concentration, the COD removal efficiency was also increased. Since COD is a component of most pollutants, therefore it can be assumed that at higher concentration of the investigated pollutant, the levels of COD increases. As a result, for TNT concentrations ranging from 1 to 30 mg/L, the average COD removal efficiency was 31.89% ± 3.03 to 35.89% ± 2.89 for 4 h and 43.67% ± 2.70 to 45% ± 2.23 for 6 h. On the other hand, it may be claimed that when pollutant concentration rises, bacteria's requirement for carbon and nitrogen to degradation and remove organic matter also rises, which may result in a rise in the effectiveness of COD removal. Additionally, when TNT content rises, bacteria require more substrate, which promotes their growth and, in turn, improves the effectiveness of COD elimination^29,31,34^.

### Determination of kinetic coefficients

One of the most crucial methods for determining the ideal conditions in terms of the process efficiency, cost-effectiveness and probable large-scale use is the determination of the kinetic coefficients and related parameters. As shown in Table 1, the kinetic coefficients are nearly constant and unchanging throughout a variety of HRT and pollutant concentrations. Only some coefficients, including K_s_, MLSS, MLVSS, and SVI, show a little variation, which is caused by variations in pollutant concentration and hydraulic retention time.

**Table 1:** Kinetic coefficients of EAAS in TNT removal.

TNT concentration	K (mg/L)	K_s_ (mg/L)	K_d_ (gr VSS/gr VSS d)	µ_max_ (/day)	MLSS (mg/L)	MLVSS (mg/L)	SVI (mL/g)	F/M (/day)	HRT (h)
1	3.16	97.17	0.07	1.15	2819.28	2246.44	142.5	0.30	4
10	3.16	96.78	0.07	1.15	2831.28	2174.67	142.94	0.35	
30	3.14	100.28	0.10	1.19	2823.17	2213.72	143.22	0.35	
1	3.16	95.36	0.07	1.15	2823.83	2249.83	143.83	0.31	6
10	3.16	96.06	0.07	1.15	2832.61	2189.06	141.28	0.34	
30	3.15	88.04	0.10	1.14	2829.61	2208.94	140.39	0.35	

### Compare removal efficiency with other studies

Table 2 summarizes the effectiveness of various biological processes in removing TNT as well as some different pollutants. Based on the reported results and the findings of the present study, biological processes using extended aeration-activated sludge shows adequate efficiency and those operated with a short processing time can be employed to remove organic pollutants. Although some of them have been carried out at longer hydraulic retention time and therefore are not typically acceptable in terms of economy and productivity.

**Table 2:** Compare TNT removal efficiency with other studies.

No	Biological process	Pollutant	Pollutant concentration (mg/L)	µ_max_ (mg/h g)	K_i_ (mg/L)	K_s_ (mg/L)	HRT (h)	Removal efficiency (%)	Refs.
1	Immobilized microorganism-biological filter	Trinitrotoluene	5	0.76	62.20	100.16	36	97.5	^35^
2	Digested sewage sludge in anaerobic condition	Trinitrotoluene	110	–	–	–	144	99	^36^
3	Continuous flow stirred-tank reactor	Trinitrotoluene	10	–	–	–	48	74	^37^
5	Extended aeration-activated sludge	Phenol	50	–	–	–	24	96	^38^
6	Extended aeration-activated sludge	Alkylbenzene Sulfonates	15.8	–	–	–	25	96.8	^39^
8	Extended aeration-activated sludge	Trinitrotoluene	30	1.14	3.15	88.05	6	84.25	This study

### Modeling using ANN

**Figure 4:**
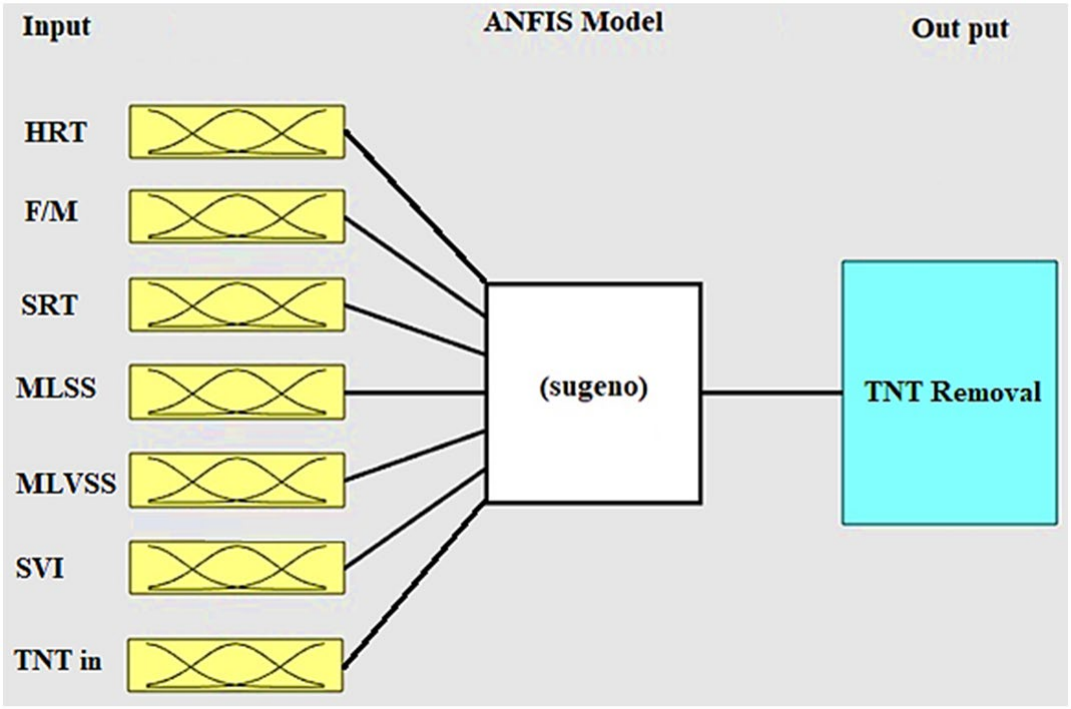
Developed ANFIS structure.

The ANFIS structure and network model were used for this work to predict ATN removal. The results are shown in Fig. 4.

As mentioned in the methods, Sugeno type FIS system and membership function were employed for carrying out the training process. The FIS has the 7 inputs of F/M, HRT, SRT, MLSS, MLVSS, SVI, and TNT_in_. In order to generate the modified rules in a certain data set, the network partitioning technique was used. For this purpose, 85% of the data set (92 samples) was used to train the ANFIS model and the remaining 15% of the data (16 samples) set was used to test the predictive proficiency of the relevant model.

The ANFIS model first performs the training process for the training data set and then tests the results with the test data. In addition, in the ANFIS model training process, the input data set was drawn several times to prevent any possible errors. The required number of iterations in order to map was stated as epochs. Also, to measure the validity and efficiency of the model after the training process, this model was tested on 16 data sets.

**Figure 5:**
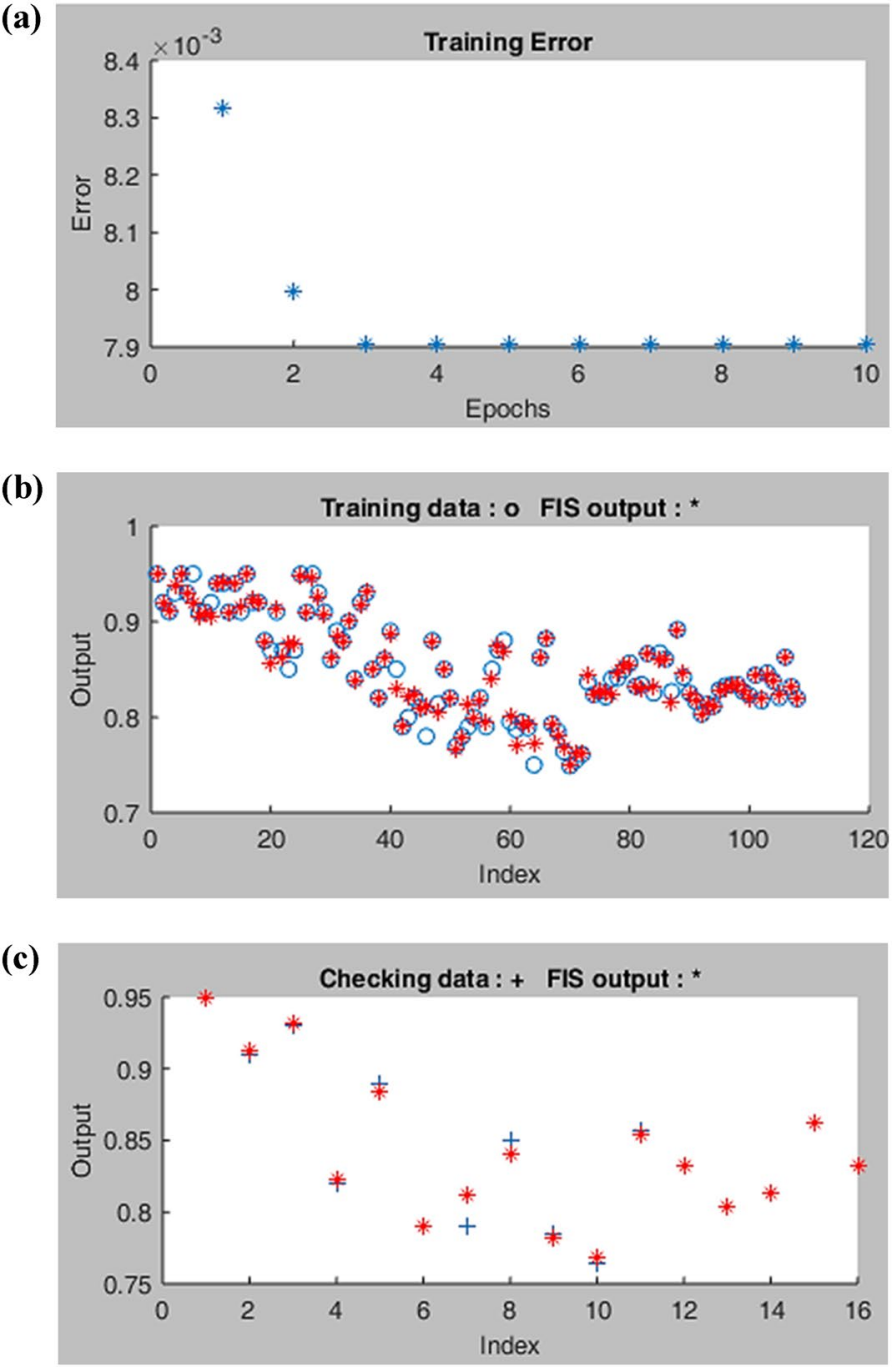
(**a**) Training error curve of ANFIS model for TNT removal, (**b**) comparison of experimental and predicted values in training, and (**c**) testing processes.

In the next step, the results obtained from the training and testing processes of the ANFIS model were analyzed by using different types of membership functions. According to the obtained results, Gaussian membership function (gaussmf) exhibited lower prediction error than other functions. The RMSE was obtained 6.593E−3 for training and 7.904E−3 for testing. ANFIS structure performed well, when 2 membership functions being assigned to each input variable. Figure 5a represents the training error curve for the experimental values of ATN removal. Figure 5b and c demonstrate the experimental results of ATN removal under similar processing conditions using the ANFIS model to compare training and experimental data sets. Moreover, *R*^2^ in this study is about 97.93% and 94.34% for the training and testing dataset, respectively, which indicates the accuracy and adequacy of the model.

### Optimization with GA

**Figure 6:**
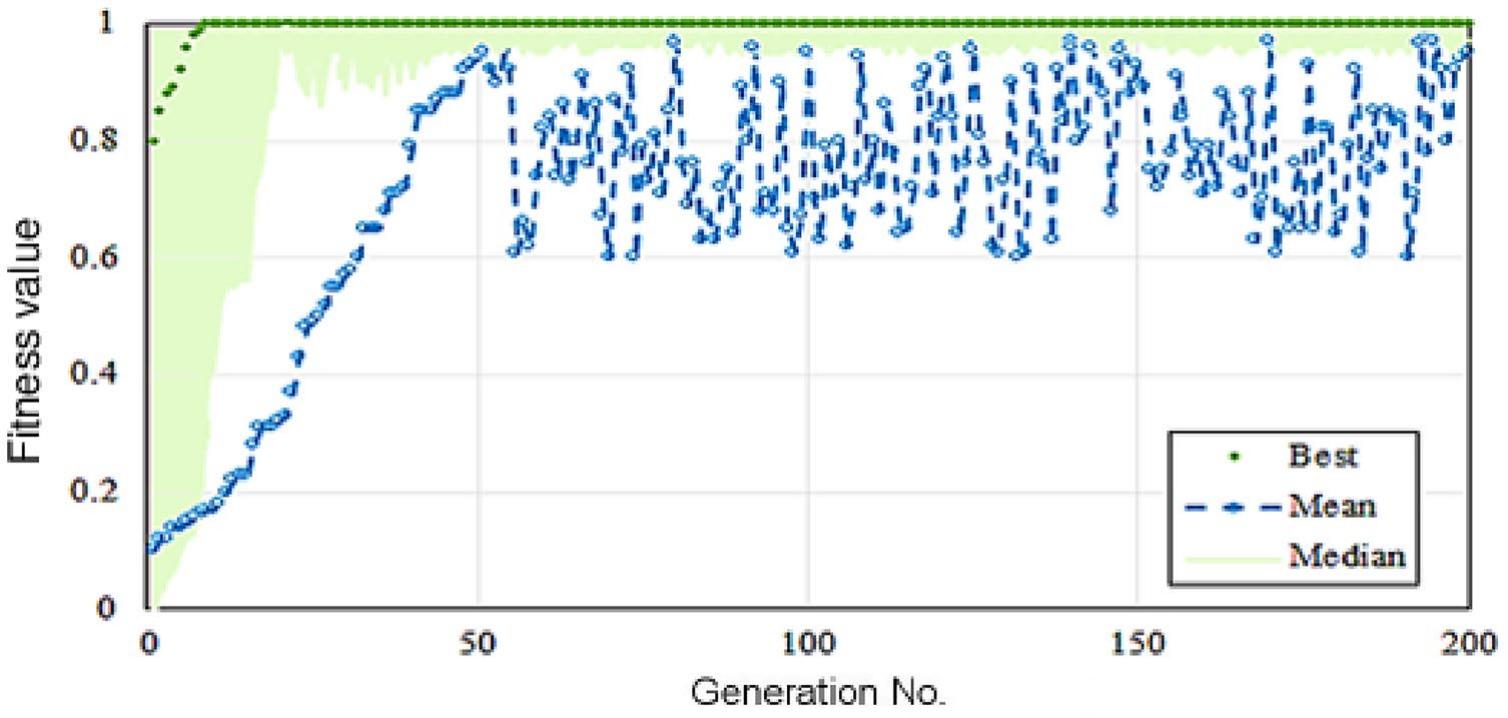
Genetic algorithm results: the best and average fitness values per generation.

In the present study, the genetic algorithm was used to optimize the input parameters and finally to achieve the highest removal efficiency. The designed ANFIS model was introduced as a fitness function to GA. In Fig. 6, a diagram of the best and average fitness values in each generation is presented. Table 3 also represents the best fitness values in the final generation. Therefore, the optimal values of the input parameters, which are F/M, HRT, SRT, MLSS, MLVSS, SVI, and TNT_in_, were equal to 0.32, 5.81, 12.12, 2814.74, 2407.21, 144.84 and 4.78, respectively, which leads to the highest removal efficiency (96.80%).

**Table 3:** Genetic algorithm results: the optimal value of the input parameters and highest removal efficiency.

Parameters	Units	Optimized value
F/M	/day	0.32
HRT	h	5.81
SRT	h	12.12
MLSS	mg/L	2814.74
MLVSS	mg/L	2407.21
SVI	mL/g	144.84
TNT_in_	mg/L	4.78
TNT removal	%	96.00

### Factors affecting the removal of TNT

**Figure 7:**
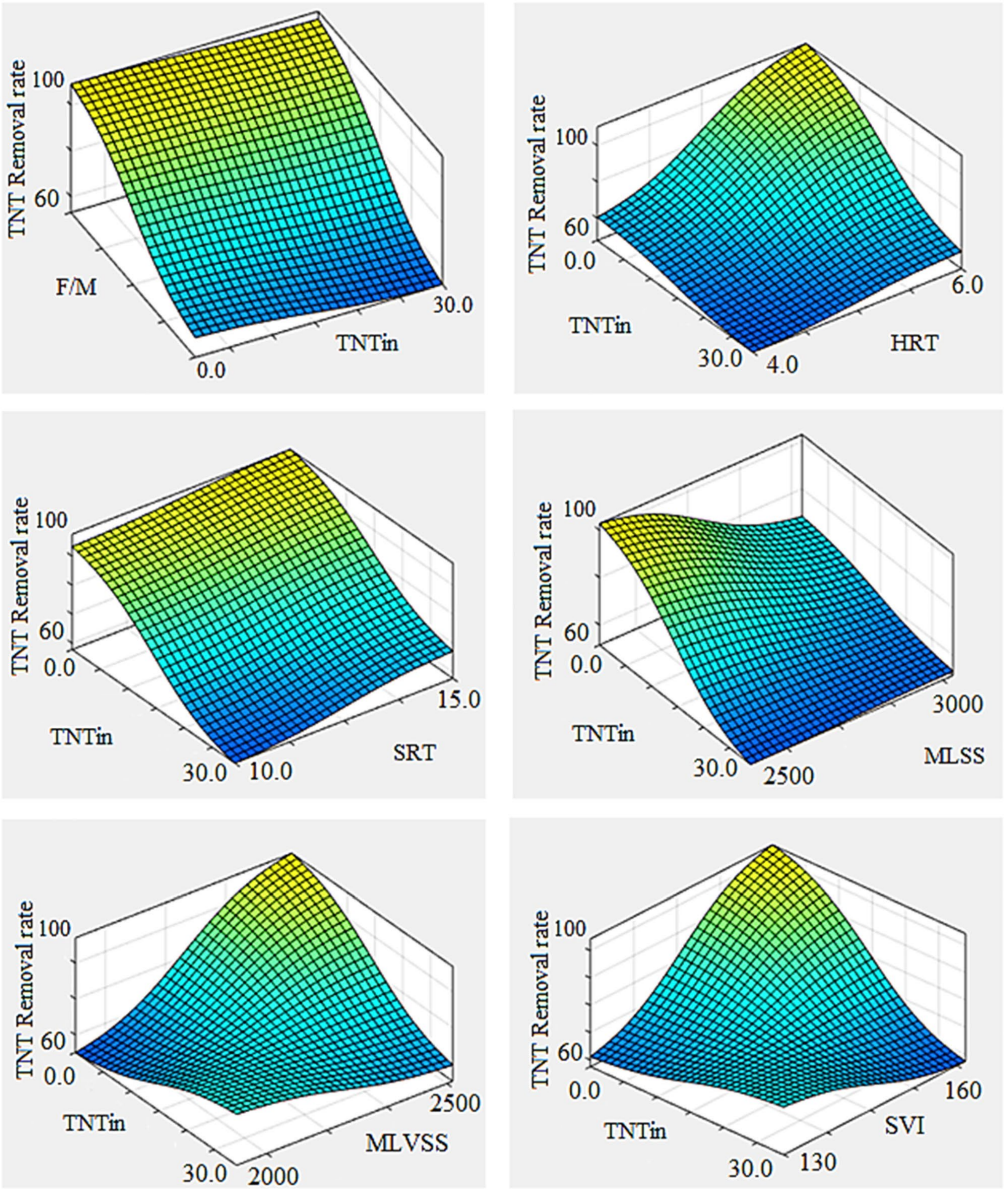
ANFIS prediction of removal rate in different input parameters.

Using the ANFIS model designed, the following diagrams were drawn in MATLAB software. The parameters shown inside each graph were considered variable and the other parameters for each graph were fixed and equal to their optimal values (obtained from the genetic algorithm) (Fig. 7).

### Sensitivity analysis

**Figure 8:**
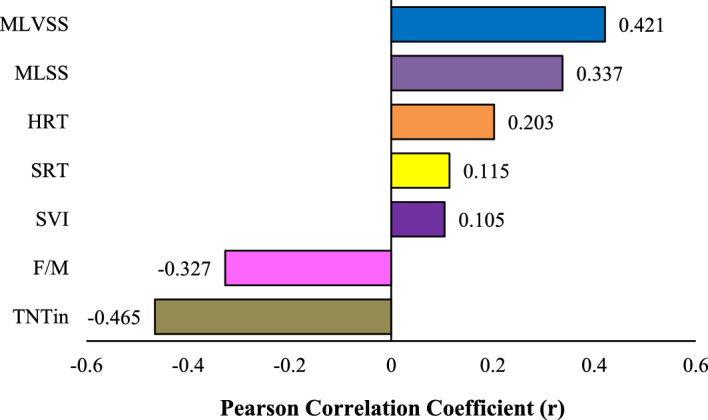
Sensitivity analysis using Pearson correlation.

Sensitivity analysis is performed to evaluate the significance of the effect of each parameter on the output variable^40^. This shows the extent to which each input factor affects the accuracy of the predicted model and finally, it is a good way to design more applications^41^. In this study, sensitivity analysis was performed by the Pearson correlation method. Based on the following diagram, the effect of the input parameters was in the order of the initial concentration of TNT_in_, MLVSS, MLSS, F/M, HRT, SRT, and SVI. The impact factor of each parameter is visible in Fig. 8.

## Conclusion

The EAAS biological process in TNT elimination was utilized in this investigation by designing a pilot with dimensions of 38.5 × 15 × 44 cm. The analyzed parameters were TNT concentration, HRT, and COD, which were optimized, and the optimal TNT removal efficiency under the circumstances of TNT 10 mg/L and HRT 6 h was 84.25%. Kinetic coefficients were computed during the TNT removal process, and they can be used subsequently for the design of the treatment plant system on a real scale. The biological processes depend on both the generation of biomass and the consumption of oxygen. These variables must be further studied with wastewaters of various compositions since they have significant effects on the design of biological wastewater treatment procedures. Additionally, the examined parameters were adjusted using artificial neural network modeling. The modeling process uses the ANFIS method and a genetic algorithm. According to the ANFIS method study, an accuracy of around 97.93% was attained. Furthermore, F/M: 0.32 /day, HRT: 5.81 h, SRT: 12.12 h, MLSS: 2814.74 mg/L, MLVSS: 2407.21 mg/L, SVI: 144.84 mL/g, TNT_in_: 4.78 mg/L, and TNT removal 96% were the values of the parameters that the genetic algorithm optimized. Consequently, this research has demonstrated the significance of optimizing TNT concentration and HRT to accomplish the process' optimal performance. For wastewaters with diverse and more complicated chemical compositions as well as nitrification/denitrification mechanisms for the removal of nitroaromatic compounds, additional research is required.

## Data Availability

The datasets used and/or analyzed during the current study available from the corresponding author on reasonable request. In addition no software were used to create images.
